# Supplementary material for: A Curriculum for Clerkship Students to Foster Professionalism Through Reflective Practice and Identity Formation
Source: MedEdPORTAL. 2016 Jun 17;12:10416. doi: 10.15766/mep_2374-8265.10416 (PMC6464454; doi:10.15766/mep_2374-8265.10416)
Supplement: Supplementary file 1 — A. Opening Session Articulating One's Ideals Facilitator's Manual.docx B. Opening Session Writing Prompt.docx C. Opening Session PowerPoint Slides.ppt D. Session Evaluation Form.docx E. Module 2 Facilitator's Guide.docx F. Module 3 Facilitator's Guide.docx G. Module 4 Facilitator's Guide.docx H. Module 4 Ideals Box Template.docx I. Module 4 Introductory Email With Table.doc [file mep-12-10416-s001.zip › F. Module 3 Facilitator's Guide.docx]

**Fostering Professionalism through Reflective Practice and Identity Formation Curriculum Module: Breaking Down Barriers**

**Facilitator’s Guide**

This is one module of the “Fostering Professionalism through Reflective Practice and Identity Formation” Curriculum series. Apart from the initial module (Articulating Your Ideals), the curriculum has been designed to allow for implementation of modules in any order and over any time period.

In this guide, we present the module entitled “Breaking Down Barriers,” which is presented during the Internal Medicine clerkship. This module is designed to allow students to delineate between internal barriers (characteristics of the individual learner) and external barriers (characteristics of the environment), and to assign a relative weight to the impact that each barrier has during training and subsequently during practice. By encouraging students to understand the ways in which barriers shift or remain constant over time, as well as recognize the presence of barriers that are under the control of the individual learner, students will be provided with some of the tools necessary to recognize future opportunities for maximizing choices and behaviors that are in accordance with their own ideals.

**Purpose and Goal of this Resource**

Purpose: Identify and delineate internal and external barriers to our adherence to our ideals in the clinical setting, understand the ways in which these barriers may shift throughout training, and identify individual approaches to clinical situations that may minimize or eliminate barriers.

Goals:

By the end of the session, each student will:

1) Describe at least one instance in which he or she did not act in accordance with their written ideals in a clinical situation.

2) List at least three barriers that prevented adherence to the ideal in this situation.

3) Define each barrier as internal (related to the student) or external (related to the team, environment, or culture).

4) Describe the weight of each barrier as it detracts from adherence to the student’s ideal.

5) Describe ways in which the number of barriers or their relative contributions might change as the student progresses through his or her medical career.

6) Using the list of barriers that has been developed, discuss with peers behavioral changes that may minimize or eliminate barriers.

**Conceptual Background for this Session**

Conceptual background for this module: This module encourages students to break the often overwhelming and intimidating array of barriers to adherence to our ideals into small, measurable, and potentially actionable items. By encouraging this type of reflection, we allow students to separate transient perceived barriers (such as the hierarchy of the medical team), from more deeply-embedded characteristics within the individuals themselves that may have a significant and long term impact on their abilities to function within their own ideals. Furthermore, by recognizing that many barriers are internal and therefore at least to some extent under the control of the individual, students can then develop modified approaches to clinical situations that allow them to overcome or minimize their own internal barriers.

**Timeline and Practical Implementation Instructions**

Overall Session Structure (90 minutes):

Introductory exercise (10 minutes):

Pose the following question to the group:

“You may have found that it is sometimes difficult to adhere closely to your ideal when you are immersed in the clinical setting. Please use your journaling time to describe an instance in which this has occurred. You may write a narrative, draw a picture, or use whatever other means are most helpful to you in order to organize your thoughts. If you would like to review your written ideal from the introductory session of this curriculum, you are free to do so. You will not be required to share either your ideal or your narrative with the group.

Once you have identified a situation in which you did not adhere fully to your ideal, please list at least three barriers that prevented you from doing so. We’re defining the term “barrier” very broadly here, so feel free to list anything, no matter how seemingly insignificant. You will be asked to share your list with the group.”

List Development and Characterization (15 minutes):

Ask the students to contribute items from their lists. The students are not required to share every item on their lists, but should be encouraged to do so. As the students share list items, ask them to characterize each item as being primarily external (related to the environment, culture, team, etc.), or primarily internal (related to the thoughts or emotions of the student). Create two master lists on a white board, projector, or chalkboard that is visible to the entire group.

Discussion of Barrier Contributions and Shifts (55 minutes):

Once both master lists have been developed, ask the students to identify barriers with weights that may change over time. For example, the desire to obtain a positive evaluation from an attending faculty member may play a significant role in the student’s behavior now, but may become less significant when the student is an attending physician. You may want to use some of the following questions in order to facilitate discussion:

• Are there any barriers that will not significantly change?

• Do these barriers have anything in common?

• Is it possible to overcome them?

• If so, how?

• What might you do differently if this situation happens again?

Debriefing (10 minutes):

Ask the students for input regarding the value and quality of the session. Reassure them that their narratives are for their own growth and are not accessible by peers or faculty. Remind them that discussion during the session should remain confidential. Provide additional debriefing for any students who were impacted emotionally during the session.

**Experience with Implementation (Tips for Deployment)**

Experience to date with this module:

- This module is facilitated by a dyad of a staff chaplain and a clinical faculty member, both of whom are skilled communicators. These individuals have expressed satisfaction with the concept of paired facilitation, which allows one facilitator to observe the group dynamic and guide discussion, while the other actively facilitates the discussion.
- Students have expressed increased understanding of the role of chaplaincy in day-to-day patient care.
- Facilitation tip: Depending on the time of year students may be more or less comfortable with what they perceive as unstructured discussion. Setting clear expectations that the session is not related to content delivery but rather discussion of professional role formation, along with the reassurance that there is no one “right” answer, can be reassuring and facilitate discussion.
