# Supplementary material for: A Curriculum for Clerkship Students to Foster Professionalism Through Reflective Practice and Identity Formation
Source: MedEdPORTAL. 2016 Jun 17;12:10416. doi: 10.15766/mep_2374-8265.10416 (PMC6464454; doi:10.15766/mep_2374-8265.10416)
Supplement: Supplementary file 1 — A. Opening Session Articulating One's Ideals Facilitator's Manual.docx B. Opening Session Writing Prompt.docx C. Opening Session PowerPoint Slides.ppt D. Session Evaluation Form.docx E. Module 2 Facilitator's Guide.docx F. Module 3 Facilitator's Guide.docx G. Module 4 Facilitator's Guide.docx H. Module 4 Ideals Box Template.docx I. Module 4 Introductory Email With Table.doc [file mep-12-10416-s001.zip › G. Module 4 Facilitator's Guide.docx]

**Fostering Professionalism through Reflective Practice and Identity Formation Curriculum Module: Identifying Forces which Impact the Ideal**

**Facilitator’s Guide**

This is one module of the “Fostering Professionalism through Reflective Practice and Identity Formation” Curriculum series. Apart from the initial module (Articulating Your Ideals), the curriculum has been designed to allow for implementation of modules in any order and over any time period.

In this guide, we present the module entitled “Identifying Forces which Impact the Ideal,” which is presented during the Underserved Medicine and Domestic Health clerkship. The purpose of this module is to enhance students’ identification of the forces (sustaining and non-sustaining) which impact the maintenance of their ideal. Also, an opportunity for reflection is provided in a neutral setting, allowing students to identify and share mechanisms which help one best sustain his/her ideal.

**Resource Files needed for this session**

1. Module 4 Introductory Email With Table (Appendix I)
2. Module 4 Ideals Box Template (Appendix H)

**Purpose and Goal of this Resource**

Purpose: Using a previously-articulated ideals statement, identify sustaining and nonsustaining forces that impact an individual’s ability to adhere to his or her ideal, and compare and contrast the relative weights of each of these forces.

Goals:

By the end of the session, each student will:

1. List sustaining and non-sustaining forces that are experienced during the clerkship.
2. Assign relative weights to each of the listed forces.
3. Describe strategies for maximizing the impact of sustaining forces and minimizing the impact of non-sustaining forces.

**Conceptual Background for this Session**:

This module is designed to foster students’ reflection about forces which impact one’s ideal. A revisiting of the ideals statement from the first module is used to anchor the reflection and activities. When this module occurs, students have had at least one clinical clerkship. As such, they have begun to feel the tension of attempting to maintain their “ideal”, while everyday forces are impacting them. However, without a deliberate mechanism to do so, they may not take the time to reflect on the situation, identify the forces, and begin to choose behaviors which help them to sustain these ideals.

This module allows the opportunity for individual reflection throughout the clerkship, by use of a table to document forces and experiences. Individual reflection to prompts at the beginning of the group session provides an additional opportunity. Finally, the group setting allows for peer interaction, shared experiences and reflection, best practices discussion, and catharsis.

**Timeline and Practical Implementation Instructions**

Students are sent an introductory email at the beginning of the first week of the clerkship (Appendix I). On the final Monday of the clerkship, students are sent a reminder with the same email. On the final Friday of the clerkship, students gather for a small group session. The room needs a white board and markers. Students bring their computers for the individual reflection component. We suggest allowing 90 minutes for this module, but it could be compressed to 60 minutes.

• On the first day of the rotation: Introductory Assignment – Students are provided with a table (Appendix I) on which they are asked to record examples of sustaining and non-sustaining forces that they encounter during the rotation. They are asked to populate the table with sustaining and non-sustaining forces during the clerkship, and bring a copy with them to the debriefing session at the end of the clerkship.

• 0-15 minutes: Individual reflection – Ask the students to choose three out of the following five prompts, and write down answers to them. While the students are writing, draw an “Ideals Box” (see Appendix H for an example) on the whiteboard.

i. Which observed preceptor behaviors or experiences from this clerkship will you emulate in your future practice as a physician? Which will you not emulate? How did these add to or change your “ideal”?

ii. Of the behaviors you observed and the experiences you had, which do you expect to have the most lasting effect on the physician you become and the way you practice medicine? Why?

iii. What is happening to you now that has the most impact on your ideal?

iv. Are you moving closer to or further from your ideal? How do you get to where you want to be?

v. How has your “ideal” evolved over this clerkship and over all of the clerkships you have completed?

• 15-30 minutes: Populating the “Ideals box.” – Ask the group to list the most important words or phrases that they included in their ideals statement. Use their responses to fill in the ideals box.

• 30-50 minutes: Charting force vectors – Ask each individual student to choose a colored marker and then chart the forces he/she recorded during the clerkship as vectors, using arrows that point towards or away from the ideals box (some forces may be bi-directional). Ask the students to graphically represent the relative magnitude of the forces by varying the thickness of the arrows.

• 50-75 minutes: Facilitated discussion – Use the prompts below to facilitate discussion. Depending on the experience of the group, it may be possible to move quickly through the first several prompts and focus most of the discussion on comparing and contrasting the impact of similar forces on different individuals, as well as strategies for maximizing sustaining forces and minimizing non-sustaining forces.

i. Which forces are elements of “the system”?

ii. Which forces are unique to the setting of your clerkship?

iii. Which types of forces did you identify/experience?

iv. Which forces did you not identify/experience, which others did?

v. Which types of forces are stronger or weaker for you? For others? What group patterns emerge?

vi. Which forces are driving the particular pattern of forces for you?

vii. How do you modify or manage the forces you perceive as having a negative impact?

viii. How do you maximize the forces you perceive as having a positive impact?

• 75-90 minutes: Debriefing - Students are asked to give input on the value of the individual session as well as the curriculum as a whole. They are encouraged to contact the facilitator or clerkship director if further discussion is needed. The students evaluate the individual module using the form in Appendix D.

**Experience with Implementation (Tips for Deployment)**

Experience to date with this module:

- This module occurs at various points in the third year curriculum as the clerkship is a four week experience, which occurs off campus. Logistically, the session is scheduled on the final day of the clerkship, because the students are at the main campus for other clerkship activities. Scheduling this activity at the end of the clerkship also gives the students the opportunity to reflect on experiences and forces which they have identified throughout the four week experience. One facilitator is present for the session.
- Students’ responses and reflection appear somewhat affected by the degree of clinical experience and where they are in the career choice process. For example, students who have had more clinical exposure often identify hierarchical relationships as a non-sustaining force.
- Some cohorts were more vocal and interactive than others. In general, participation was adequate and there was not a tendency for one or two students to control the group function. However, at times, one or two themes would be the main focus of reflection and discussion (e.g. debt, the uncertain future of healthcare).
