# Supplementary material for: A Curriculum for Clerkship Students to Foster Professionalism Through Reflective Practice and Identity Formation
Source: MedEdPORTAL. 2016 Jun 17;12:10416. doi: 10.15766/mep_2374-8265.10416 (PMC6464454; doi:10.15766/mep_2374-8265.10416)
Supplement: Supplementary file 1 — A. Opening Session Articulating One's Ideals Facilitator's Manual.docx B. Opening Session Writing Prompt.docx C. Opening Session PowerPoint Slides.ppt D. Session Evaluation Form.docx E. Module 2 Facilitator's Guide.docx F. Module 3 Facilitator's Guide.docx G. Module 4 Facilitator's Guide.docx H. Module 4 Ideals Box Template.docx I. Module 4 Introductory Email With Table.doc [file mep-12-10416-s001.zip › H. Module 4 Ideals Box Template.docx]

IDEAL

| **As a group exercise, the students begin by populating the box on a white board with words and phrases which represent the “ideal physician”.** |
| --- |

**After the box is completed above, students diagram the sustaining and non-sustaining forces which affect their “ideal.” Vectors (magnitude and direction), as well as colors of the student’s choice, are used to represent the forces. Some forces are bidirectional.**
