# Supplementary material for: A Curriculum for Clerkship Students to Foster Professionalism Through Reflective Practice and Identity Formation
Source: MedEdPORTAL. 2016 Jun 17;12:10416. doi: 10.15766/mep_2374-8265.10416 (PMC6464454; doi:10.15766/mep_2374-8265.10416)
Supplement: Supplementary file 1 — A. Opening Session Articulating One's Ideals Facilitator's Manual.docx B. Opening Session Writing Prompt.docx C. Opening Session PowerPoint Slides.ppt D. Session Evaluation Form.docx E. Module 2 Facilitator's Guide.docx F. Module 3 Facilitator's Guide.docx G. Module 4 Facilitator's Guide.docx H. Module 4 Ideals Box Template.docx I. Module 4 Introductory Email With Table.doc [file mep-12-10416-s001.zip › _Educational Summary Report_10416.pdf]

MedEdPORTAL®
The Journal of Teaching and Learning Resources

Original Publication

OPEN ACCESS

A Curriculum for Clerkship Students to Foster Professionalism Through Reflective Practice and Identity Formation

Susan A. Glod, MD, MEd*, David Richard, MD, Patricia Gordon, MD, Mary Lynn Fecile, MD, PhD, Deborah Kees-Folts, MD, Margaret Kreher, MD, Eileen M. Moser, MD, MHPE, Daniel R. Wolpaw, MD, Chengwu Yang, MD, MS, PhD, Paul Haidet, MD, MPH
*Corresponding author: sglod@hmc.psu.edu

Abstract

Introduction: Research suggests that students become less patient-centered and empathetic in response to both internal and external factors, including the organizational culture, or hidden curriculum, of medical school. Students often feel compelled to make compromises when they experience tension between competing values in clinical teaching environments. To address this, we implemented a modular, longitudinal professionalism curriculum for third-year medical students, based on a conceptual model that highlights a student's ideal, as well as the internal and environmental forces that can either sustain or change their ideal over time. **Methods:** As students progressed through the third year, they participated in various modules linked to different clerkships, each focusing on a different aspect of the conceptual model. Each module includes a reflective writing exercise followed by a faculty-facilitated discussion. **Results:** In general, students rated the group discussions and faculty facilitation as the most useful parts of each session and the writing exercises as the least useful. Written comments were mostly favorable and suggested that the session facilitated self-reflection and provided a safe environment for students to discuss stressors of third-year clerkships. **Discussion:** This curriculum represents a unique approach to fostering professional role formation through its broad potential applicability to multiple types and levels of learners, its adaptability to fit various course lengths and learning environments, and its incorporation of a conceptual model that allows individual learners to address different facets of the sustaining and acculturating forces that impact their personal professional identity formation for future encounters.

Keywords

Communication, Reflective Practice, Ideals, Cognitive Dissonance, Identity Formation, Role Formation

Citation: Glod SA, Richard D, Gordon P, et al. A curriculum for clerkship students to foster professionalism through reflective practice and identity formation. *MedEdPORTAL*. 2016;12:10416. https://doi.org/10.15766/mep_2374-8265.10416

Copyright: © 2016 Glod et al. This is an open-access article distributed under the terms of the Creative Commons Attribution license.

Appendices

A. Opening Session Articulating One's Ideals Facilitator's Manual.docx
B. Opening Session Writing Prompt.docx
C. Opening Session PowerPoint Slides.ppt
D. Session Evaluation Form .docx
E. Module 2 Facilitator's Guide .docx
F. Module 3 Facilitator's Guide .docx
G. Module 4 Facilitator's Guide .docx
H. Module 4 Ideals Box Template.docx
I. Module 4 Introductory Email With Table.doc

All appendices are peer reviewed as integral parts of the Original Publication.

Educational Objectives

Upon completing the curriculum, learners will be able to:

1. State their ideals for practicing medicine while comparing and contrasting these with formal statements by the profession.
2. Express, through written reflection, how their ideals were challenged during clinical rotations.
3. Apply appreciative inquiry to reconstruct the scenario from the clinical rotation.
4. Identify internal and external barriers to their adherence to their ideals in the clinical setting.
5. Identify individual approaches to clinical situations that may minimize or eliminate these barriers.

Introduction

Research suggests that students become less patient-centered in response to the organizational culture, or hidden curriculum, of medical school.[1] Students often feel compelled to make compromises when they experience tension between competing values in clinical teaching environments.[2,3] The recognition that

each student enters the medical school training environment with a set of personal experiences and encounters that differs from student to student necessarily leads to potential dissonance when clinical encounters challenge a developing professional identity. Given the varying development of each student's identity formation, clinical encounters that challenge these underpinnings might lead to differing degrees of discomfort and tension in the clinical setting. Allowing for a curricular rest stop for self-reflection and facilitated discussion in a purposeful, longitudinal, and safe curricular environment might give an individual learner an opportunity to recalibrate an evolving identity removed from the acute clinical scenario that led to the dissonance. To address this, we implemented a modular, longitudinal professionalism curriculum for third-year medical students.

Our curriculum is guided by a model (see Figure) that considers the tension created when a student encounters behaviors and attitudes that differ from what he/she considers to be ideal; this model is based on theories of cognitive dissonance.[4] Our reflective activities focus on helping students to (a) individually define and articulate their own ideals regarding medical practice, (b) recognize forces in medical care that may either pull them away from or help them to sustain their own ideals, and (c) make deliberate choices about their own behaviors in the face of sustaining and acculturating forces as they encounter various organizational cultures in clinical care.

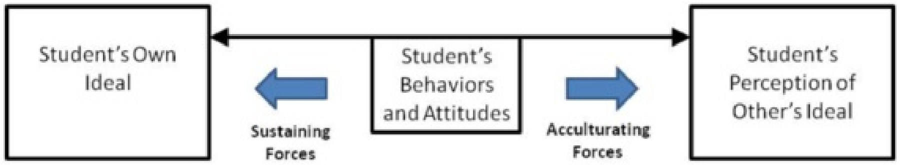

Figure. Conceptual model of forces impacting students' behaviors and attitudes.

Our target audience was third-year clerkship students as they progressed through their clinical rotations. Our school holds a class-wide intersession that occurs immediately prior to the clerkship year. We embedded the initial session within this intersession. In doing so, we were able to introduce the entire class to the goals and structure of the curriculum before the students dispersed to various clerkships. It was also our goal to foster reflection on the students' individual ideals before they were exposed to clinical rotations.

Methods

Our curriculum consists of four modules, each comprising a set of classroom activities. The module entitled Articulating One's Own Ideals is intended to occur first in the sequence. The three subsequent modules can occur in any order. We tied each module to a particular clerkship (family medicine, internal medicine, and underserved medicine), meaning that individual students experienced the modules in varying order and timing based on their clerkship schedules. However, the modules are designed to exist independent of discipline and so could occur at any point(s) in the third year.

We conducted the opening module with 150 students (our entire class) immediately before beginning clinical clerkships and then conducted subsequent modules with small groups of students (six to 15) during the clerkships noted above. We designed module procedures based on the class sizes we encountered; however, the procedures for any given module can be easily adapted to accommodate between six and 200 learners. For example, because we conducted the opening module with 150 students, it uses PowerPoint slides (Appendix C) to aid in orientation to the session tasks. We did not include PowerPoint slides for subsequent modules but did use flip charts, blackboards, and other writing/display tools to accommodate the smaller-sized groups in these venues.

All of the modules are designed to occur as single hour-long educational sessions, with no pre- or postsession homework. Each session begins with a 10- to 15-minute individual writing prompt, followed by 45-50 minutes of group discussion. The methods for fostering group discussion and the writing prompts vary from module to module.

Module 1 (Opening Module): Articulating One's Own Ideals

The opening module is designed to foster students' reflection about and clear articulation of an individual ideals statement. We believe the ideals statement to be foundational to future reflection on and in action, because it provides students with an individual anchor to help guide choices when they find themselves in challenging situations during clinical care. The ideals statement is the foundation upon which subsequent modules in the curriculum build.

We suggest using 90 minutes for this portion of the curriculum, although the session could be abbreviated to 60 minutes by reducing the time for small- and large-group discussion during the pair-share segments. Detailed instructions are provided in the individual facilitator's guide (Appendix A), and in the notes section of the Articulating One's Own Ideals PowerPoint slide deck (Appendix C), but a general time line for the session is as follows:

- 0-15 minutes (Slides 1-6): introduction—part of the introduction depicts a scenario where a student witnesses physicians engaging in unprofessional behavior in front of a patient.
- 15-30 minutes (Slides 7-8): individual writing assignment—during this portion of the session, students are given a writing prompt (Appendix B) that asks them to articulate their own individual vision of what they want to be like as a physician.
- 30-55 minutes (Slide 9): pair-share process—10 minutes to discuss in pairs, 15 minutes to debrief as a large group facilitated by the instructor. During this portion of the session, students are asked to compare and contrast their individual visions with partners and then with the group.
- 55-60 minutes (Slide 10): class reads the Modern Hippocratic Oath out loud.
- 60-85 minutes (Slides 11-12): second pair-share task and orientation to the rest of the modules—during this portion of the session, pairs are asked to reflect on the scenario presented in the introduction and describe what they would realistically do if faced with a similar scenario. The session finishes with an overview of the different curricular pieces that the students will encounter in different clerkships.
- 85-90 minutes (Slide 13): evaluation—the students evaluate the individual module; our evaluation tool is included in Appendix D.

Module 2: When Your Ideals Are Challenged, Does Your Anchor Shift?

This module is designed to encourage students to reflect upon their core belief structure and to analyze the acculturating forces that may challenge those beliefs. By allowing students the opportunity to articulate how their prior ideals, individually attained and shaped through prior experiences and socialization, have been challenged in the medical setting, they will be able to explore alternative viewpoints for future encounters. Such reflection may further strengthen a belief structure or allow mild modification in future encounters by an understanding of how others might view a similar scenario. In either case, such reflection will help the student better approach such occurrences in the future by allowing a review of the clinical situation from a personal and global viewpoint.

We implemented this module during a small-group session in the family medicine clerkship, although the module was designed to allow for implementation during any clerkship. Each session was attended by approximately 15 students (those rotating through the family medicine clerkship that month) and facilitated by one faculty member. We suggest allowing 60 minutes for this module. Detailed implementation instructions are provided in the individual facilitator's guide (Appendix E), but a general time line for the session is as follows:

- 0-15 minutes: writing exercise—learners are asked to write about an experience during which their ideals were challenged by other individuals. It is made clear to the students that their reflective writing is for their individual use only and that each student may choose not to share the writing with other students or faculty.
- 15-30 minutes: pair-share task—learners are asked to discuss the scenarios in dyads and are provided with a list of prompting questions, such as "How did the encounter change your perspective OR help anchor your beliefs?"

- 30-50 minutes: facilitated discussion—the faculty facilitator invites those who wish to share to describe their experiences and encourages discussion of similar experiences of other students. Facilitators guide discussion towards reflection on how members of the group might react to similar scenarios in the future.
- 50-60 minutes: debriefing—students are asked to give input on the value of the individual session as well as the curriculum as a whole. They are encouraged to contact the facilitator or clerkship director if further discussion is needed. The students complete the written evaluation included in Appendix D.

Module 3: Breaking Down Barriers

This module encourages students to break the often overwhelming and intimidating array of barriers to adherence to ideals down into small, measurable, and potentially actionable items. By encouraging this type of reflection, we allow students to separate transient perceived barriers (such as the hierarchy of the medical team) from more deeply embedded characteristics within individuals themselves that may have a significant and long-term impact on their ability to function within their own ideals. Furthermore, by recognizing that many barriers are internal and therefore at least to some extent under the control of the individual, students can then develop modified approaches to clinical situations that allow them to overcome or minimize their own internal barriers.

We implemented this module during a small-group session in the internal medicine clerkship, although the module can be implemented during any clerkship. Each session was attended by approximately 15 students (those rotating through a subspecialty medicine rotation) and cofacilitated by a faculty member and a member of the hospital chaplaincy staff. We suggest 75 minutes for this module. Detailed implementation instructions are included in the individual facilitator's guide (Appendix F), but a general time line for the session is as follows:

- 0-10 minutes: writing exercise—learners are asked to write about an experience during which they did not adhere to their ideal in the clinical setting and to compose a list of barriers that prevented them from doing so. We did not set any limitations on the definition of the term *barrier* and asked the students to define this as broadly as they wished.
- 10-25 minutes: list development and characterization—the facilitator asks the students to name the barriers they listed. They are not required to share every barrier on their lists but are encouraged to contribute at least one. Students are asked to decide whether or not the barrier is external to the student (related to the environment, culture, team, etc.) or internal to the student (related to the student's thoughts, emotions, values, etc.). The facilitator makes two master lists on a whiteboard.
- 25-75 minutes: facilitated discussion—the facilitator asks the students to describe the ways in which the weights of barriers may change over time. For example, the desire to get an honors grade may have a heavy weight for a student, but the weight may diminish over time as the learner moves through residency and becomes a faculty member. The barrier of being called from the bedside to complete discharge paperwork may have little weight for students but may weigh more as they progress into residency. The learners are then asked to reflect on whether or not it is possible to overcome any of the barriers they have listed and how this could be accomplished.
- 75-90 minutes: debriefing—students are asked to give input on the value of the individual session as well as the curriculum as a whole. They are encouraged to contact the facilitator or clerkship director if further discussion is needed. The students complete the written evaluation included in Appendix D.

Module 4: Identifying Forces That Impact the Ideal

This module is designed to foster students' reflection about forces that impact one's ideal. A revisiting of the ideals statement from the first module is used to anchor the reflection and activities. At the beginning of the rotation, the students are provided with a template on which they are asked to document sustaining and nonsustaining forces that impact their ability to adhere to their ideal. In a group session at the conclusion of the rotation, the group shares experiences and reflection and has a discussion of best practices.

We implemented this module during a small-group session in the underserved medicine and domestic health clerkship, although the module was designed to allow for implementation during any clerkship. Each session was attended by approximately 15 students (those rotating through the family medicine clerkship that month) and facilitated by one faculty member. We suggest allowing 90 minutes for this module, although it could be compressed into 60 minutes. Detailed implementation instructions are provided in the individual facilitator's guide (Appendix G), but a general time line for the session is as follows:

On the first day of the rotation: introductory assignment—students are provided with a table (Appendix I) on which they are asked to record examples of sustaining and nonsustaining forces that they encounter during the rotation. They are asked to populate the table with sustaining and nonsustaining forces during the clerkship and to bring a copy with them to the debriefing session at the end of the clerkship.

- 0-15 minutes: individual reflection—students are asked to take this time to choose three out of the five available prompts and write down their answers. While they are composing answers to the prompts, the facilitator prepares an ideals box schematic (see Appendix H for an example) on a whiteboard.
- 15-30 minutes: populating the ideals box—using their previously written ideals statements, the group populates the box with words or phrases that represent their ideals.
- 30-50 minutes: charting force vectors—each individual student is asked to chart the forces he/she recorded during the clerkship as vectors, using arrows that point towards or away from the ideals box (some forces may be bidirectional). The students are asked to graphically represent the relative magnitude of the forces by varying the thickness of the arrows.
- 50-75 minutes: facilitated discussion—using prompts provided in the facilitator's manual, students participate in facilitated discussion that focuses on identifying strategies for minimizing the impact of nonsustaining forces and maximizing the impact of sustaining forces.
- 75-90 minutes: debriefing—students are asked to give input on the value of the individual session as well as the curriculum as a whole. They are encouraged to contact the facilitator or clerkship director if further discussion is needed. The students evaluate the individual module using the form in Appendix D.

Results

Following each session, we surveyed the participants using a six-item survey in traditional Likert format. Each of the items used a 7-point scale except for item 6, which used a 6-point scale. We also asked students to submit qualitative comments for each session, the results of which are summarized below.

Module 1: Articulating One's Own Ideals
One hundred twenty-nine out of approximately 150 students completed the survey.

1. Please rate how easy it was for you to reflect on your experiences in today's session (4.80).
2. Please rate the quality of your reflective process during today's session (4.78).
3. Please rate the usefulness of the individual assignment (writing) during today's session (5.05).
4. Please rate the usefulness of the group discussions during today's session (4.62).
5. Please rate the helpfulness of the faculty facilitator during today's session (5.33).
6. Please rate your agreement with this statement: "I feel like I have new ideas to try out after today's session" (4.62).

Most qualitative comments for this session focused on its problematic timing; the session was held at the end of a full day of lecture/compliance training for students about to begin clinical clerkships. Some students expressed interest in the concepts that were introduced during the session but felt too tired or burned out to fully participate. There was also one comment that we feel demonstrates the reach of the clinical years' hidden curriculum even before the students had started ward rotations:

- This session was really well-intentioned, but honestly we're all more concerned about screwing up and killing someone rather than losing empathy.

Module 2: When Your Ideals Are Challenged, Does Your Anchor Shift?
Session evaluation averages on a 7-point Likert scale for 2 years (*n* = 192) are as follows:

1. Please rate how easy it was for you to reflect on your experiences in today's session (5.43).
2. Please rate the quality of your reflective process during today's session (5.24).
3. Please rate the usefulness of the individual assignment (writing) during today's session (4.63).
4. Please rate the usefulness of the group discussions during today's session (5.60).
5. Please rate the helpfulness of the faculty facilitator during today's session (6.18).
6. Please rate your agreement with this statement: "I feel like I have new ideas to try out after today's session" (4.76).

Qualitative comments expressed appreciation for the opportunity to self-reflect on challenging situations. Students found that discussing situations within the group could either facilitate reflection, for example, when other students shared their responses to similar situations, or hinder reflection, for example, when other students were less engaged in the session.

Module 3: Breaking Down Barriers
Session evaluation averages on a 7-point Likert scale for 2 years (*n* = 192) are as follows:

1. Please rate how easy it was for you to reflect on your experiences in today's session (5.38).
2. Please rate the quality of your reflective process during today's session (5.24).
3. Please rate the usefulness of the individual assignment (writing) during today's session (4.43).
4. Please rate the usefulness of the group discussions during today's session (5.41).
5. Please rate the helpfulness of the faculty facilitator during today's session (5.57).
6. Please rate your agreement with this statement: "I feel like I have new ideas to try out after today's session" (4.94).

Student comments identified the opportunity for group discussion in a safe environment as a positive aspect of this session. It is worth noting that this session was not facilitated by a clerkship director but by a separate faculty member who was not directly or indirectly involved in the clerkship assessment process. One comment suggested that the curriculum be implemented for residents and faculty.

Module 4: Identifying Forces That Impact the Ideal
Session evaluation averages on a 7-point Likert scale for 2 years (*n* = 192) are as follows:

1. Please rate how easy it was for you to reflect on your experiences in today's session (5.55).
2. Please rate the quality of your reflective process during today's session (5.63).
3. Please rate the usefulness of the individual assignment (writing) during today's session (4.64).
4. Please rate the usefulness of the group discussions during today's session (5.94).
5. Please rate the helpfulness of the faculty facilitator during today's session (6.17).
6. Please rate your agreement with this statement: "I feel like I have new ideas to try out after today's session" (5.41).

Students wrote a number of comments about the helpfulness of the schematic used during this session, which allowed them to better visualize and conceptualize sustaining and nonsustaining forces. Students also appreciated discussions within their peer groups. This session was held immediately after the final exam for the clerkship, and some students commented that the fatigue they felt following the exam hampered their ability to fully engage in the exercise.

Discussion

Our experience to date with the entire curriculum (four modules implemented over 1 year) suggested the following thoughts and reflections.

The curriculum is intended to be modular, meaning that with the exception of the first module (where students articulate their own practice ideals), the rest of the modules were designed to occur in any order at any point during the clinical years. At Penn State, we managed the logistics of the curriculum by assigning one module to each of three participating core clerkships (family medicine, internal medicine, and underserved medicine). This means that individual students experienced the modules in different orders and with different timings, depending on their individual clerkship schedules. Future work with this curriculum should explore the advantages and disadvantages of different models of delivery with respect to curricular context and timing. One of the disadvantages of our scheduling model is that some students had long periods of time between sessions, leading to extinguishment of some of the concepts of the curriculum; we are brainstorming ways of delivering the curriculum in more regular and predicable fashion in order to keep the ideas fresh in students' minds.

The clerkship directors from each of the assigned clerkships were active participants in curricular design and project development. Although each clerkship director acknowledged that time within the clerkship was a precious resource, all of them expressed commitment to the idea that professional role formation is an important aspect of the third-year curriculum warranting dedicated curricular time. Clerkship directors worked creatively to embed the sessions in a way that minimized disruptions to the rest of the rotation. For example, some clerkships made use of the lunch hour to implement the sessions, while others tacked the session before or after another required clerkship group activity.

We purposely designed this curriculum to be ungraded and to not have any student assessments (other than required attendance). Our rationale for this decision was to foster free discussion of ideas and to allow students to give voice to both positive and negative experiences, particularly in sessions where the core clerkship director was present (the clerkship directors were part of our pool of facilitators). For the most part, we found students to be very open and frank in their discussions. One of the downsides of this decision, though, was that participation was variable across students, with facilitators often estimating about 10% of students to have low engagement across sessions.

While the modules in the curriculum have unique ways of fostering student discussion, all have a common overall structure of 15 minutes of individual writing followed by 45 minutes of group discussion. We chose to begin each session with a writing assignment for two reasons: (a) we found that writing provided a good trigger for subsequent discussion, and (b) the temporal relationship of writing immediately followed by discussion worked better (in terms of fostering discussion and not having to police the writing assignments) than requiring advance writing prior to coming to the session. We noticed that students who had access to writings that they had already done in the curriculum had enhanced participation in subsequent curriculum modules; however, most students either forgot or lost the writings from prior sessions. We therefore created an electronic folder in our course management system for each individual student (which only the student could access) in order to keep students' documents in an easily accessible and secure place. We reminded students to bring their laptops or Internet-connected devices to the sessions so that they could complete and save the writing assignments into their unique folders.

Some notes on facilitation of the modules in this curriculum: Facilitators need to be comfortable with letting the conversation flow and with probing students to deeper levels of reflection. They need to have skill in helping students not try to fix each other's problems but, rather, draw out the collective wisdom of the group or classroom. We used a variety of facilitation models across the modules (e.g., clerkship director as facilitator, cofacilitation with hospital chaplains, multiple discipline-based facilitators, etc.). Each model had strengths and drawbacks, but none emerged as better or worse than the others.

Susan A. Glod, MD, MEd: Assistant Professor, Department of Medicine, Penn State Milton S. Hershey Medical Center

David Richard, MD: Associate Professor, Department of Family Medicine, Pennsylvania State University

Patricia Gordon, MD: Associate Professor, Department of Pediatrics, Pennsylvania State University College of Medicine

Mary Lynn Fecile, MD, PhD: Assistant Professor, Department of Pediatrics, Pennsylvania State University College of Medicine

Deborah Kees-Folts, MD: Professor, Department of Pediatrics, Pennsylvania State University College of Medicine

Margaret Kreher, MD: Associate Professor, Department of Medicine, Pennsylvania State University College of Medicine

Eileen M. Moser, MD, MHPE: Associate Dean for Medical Education, Office of Medical Education, Pennsylvania State University College of Medicine; Associate Professor, Department of Medicine, Pennsylvania State University College of Medicine

Daniel R. Wolpaw, MD: Professor, Departments of Medicine and Humanities, Pennsylvania State University College of Medicine

Chengwu Yang, MD, MS, PhD: Assistant Professor, Department of Public Health Sciences, Pennsylvania State University; Measurement Specialist, Office for Scholarship in Learning and Education Research, Pennsylvania State University

Paul Haidet, MD, MPH: Director of Medical Education Research, Pennsylvania State University College of Medicine; Co-Director, Office for Scholarship in Learning and Education Research, Pennsylvania State University College of Medicine; Professor, Departments of Medicine, Humanities, and Public Health Sciences, Pennsylvania State University College of Medicine

Disclosures
None to report.

Funding/Support
This work was made possible through the Edward D. Harris Professionalism Award from the Alpha Omega Alpha Honor Medical Society.

Ethical Approval
This publication contains data obtained from human subjects and received ethical approval.

References

1. Inui T. *A Flag in the Wind: Educating for Professionalism in Medicine*. Washington, DC: Association of American Medical Colleges; 2003.
2. Cohn FG, Shapiro J, Lie DA, Boker J, Stephens F, Leung LA. Interpreting values conflicts experienced by obstetrics-gynecology clerkship students using reflective writing. *Acad Med.* 2009;84(5):587-596. http://dx.doi.org/10.1097/ACM.0b013e31819f6ecc
3. Caldicott CV, Faber-Langendoen K. Deception, discrimination, and fear of reprisal: lessons in ethics from third-year medical students. *Acad Med.* 2005;80(9):866-873. http://dx.doi.org/10.1097/00001888-200509000-00018
4. Thompson BM, Teal CR, Rogers JC, Paterniti DA, Haidet P. Ideals, activities, dissonance, and processing: a conceptual model to guide educators' efforts to stimulate student reflection. *Acad Med.* 2010;85(5):902-908. http://dx.doi.org/10.1097/ACM.0b013e3181d7423b

Received: September 23, 2015 | **Accepted:** May 13, 2016 | **Published:** June 17, 2016
