# Supplementary material for: A Curriculum for Clerkship Students to Foster Professionalism Through Reflective Practice and Identity Formation
Source: MedEdPORTAL. 2016 Jun 17;12:10416. doi: 10.15766/mep_2374-8265.10416 (PMC6464454; doi:10.15766/mep_2374-8265.10416)
Supplement: Supplementary file 1 — A. Opening Session Articulating One's Ideals Facilitator's Manual.docx B. Opening Session Writing Prompt.docx C. Opening Session PowerPoint Slides.ppt D. Session Evaluation Form.docx E. Module 2 Facilitator's Guide.docx F. Module 3 Facilitator's Guide.docx G. Module 4 Facilitator's Guide.docx H. Module 4 Ideals Box Template.docx I. Module 4 Introductory Email With Table.doc [file mep-12-10416-s001.zip › A. Opening Session Articulating One's Ideals Facilitator's Manual.docx]

**Fostering Professionalism through Reflective Practice and Identity Formation Curriculum Module: Articulating One’s Own Ideals**

**Facilitator’s Guide**

Research suggests that students become less patient-centered in response to the organizational culture, or “hidden curriculum,” of medical school. Students often feel compelled to make compromises when they experience tension between competing values in clinical teaching environments. To address this, we implemented a modular, longitudinal professionalism curriculum for third-year medical students.

Our curriculum, called the “Fostering Professionalism through Reflective Practice and Identity Formation” Curriculum, consists of 4 modules, each consisting of a set of classroom activities. The opening module (“Articulating One’s Own Ideals”) is intended to occur first in the sequence. The three subsequent modules can occur in any order. We tied each module to a particular clerkship (family medicine, internal medicine, and underserved medicine), meaning that individual students experienced the modules in varying order and timing, based on their clerkship schedules. However, the modules are designed to exist independent of discipline, so could occur at any point(s) in the third year.

We conducted the opening module with 150 students (our entire class) immediately before beginning clinical clerkships, and then conducted subsequent modules with small groups of students (6-15) during the clerkships noted above. We designed module procedures based on the class sizes we encountered, however, the procedures for any given module can be easily adapted to accommodate between 6 and 200 learners. For example, because we conducted it with 150 students, the opening module uses PowerPoint slides to aid in orientation to the session tasks. We did not include PowerPoint slides for subsequent modules, but did use flip-charts, blackboards, and other writing/display tools to accommodate the smaller sized groups in these venues.

All of the modules are designed to occur as single hour-long educational sessions, with no pre- or post-session homework. Each session begins with a 10-15 minute individual writing prompt, followed by 45-50 minutes of group discussion. The methods for fostering group discussion and the writing prompts vary from module to module.

In this guide, we present the opening module in the “Fostering Professionalism through Reflective Practice and Identity Formation” Curriculum. We typically conduct this module with the entire medical student class 1-2 weeks before they start their clinical clerkships, but it could be conducted anytime during the preclerkship curriculum. This module is intended to help students to articulate their own ideals for medical practice. These ideals will be used in subsequent modules to help students brainstorm, plan, and implement ideas that will help them to act in accordance with their own ideals in situations where they might feel pressure to do otherwise.

**Resource Files included in the Submission**

1. Opening session PowerPoint slides (Appendix C)
2. Opening session writing prompt (Appendix B)
3. Opening session evaluation form (Appendix D)

**Purpose and Goal of this Resource**

Purpose: To reflect on and formally state one’s ideals for practicing medicine, and compare and contrast these with formal statements by the profession.

Goals: At the end of this module, students should be able to:

1. Articulate their own medical practice ideal
2. Compare and contrast their own ideal with current professionalism statements
3. Make choices that, in real time, are consistent with their own ideals

**Conceptual Background for this Session**


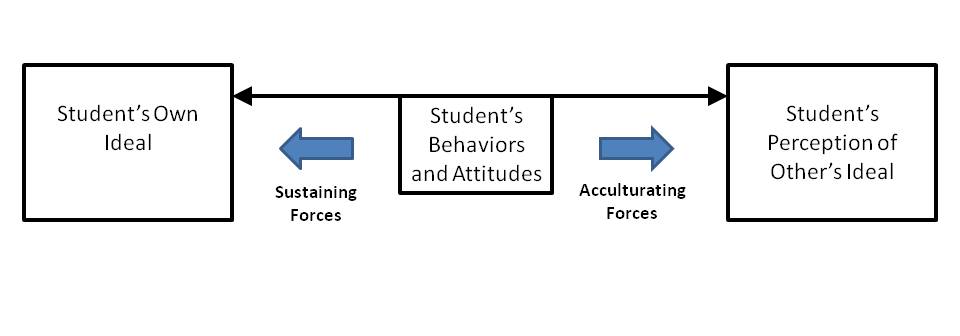
Conceptual background for the curriculum: Our curriculum is guided by a model (Figure 1) that considers the tension created when a student encounters behaviors and attitudes that differ from what they consider to be ideal, and is based on theories of cognitive dissonance.^1^ Our reflective activities focus on helping students to: a) individually define and articulate their own ideals regarding medical practice, b) recognize forces in medical care that may either pull them away from or help them to sustain their own ideals, and c) make deliberate choices about their own behaviors in the face of sustaining and acculturating forces as they encounter various organizational cultures in clinical care.

Conceptual background for this module: As the opening to the curriculum, this module is designed to foster students’ reflection about and clear articulation of an individual ideals statement. We believe the ideals statement to be foundational to future reflection on- and in-action, because it provides each student with an individual “anchor” to help guide choices when they find themselves in challenging situations during clinical care. The ideals statement is the foundation upon which subsequent modules in the Penn State Reflective Practice and Identity Formation Curriculum build.

**Timeline and Practical Implementation Instructions**

Detailed slide-by-slide instructions (with timings) are included in the “notes pages” view in the powerpoint slide deck (Appendix C). We would suggest 90 minutes for this session. If that amount of time were not available, the session could be abbreviated to 60 minutes by reducing the time for small and large group discussion during the pair/share segments. A general timeline for the session follows:

- 0-15 minutes: Slides 1-6: introduction
- 15-30 minutes: Slides 7-8: Individual Writing Assignment (Appendix B)
- 30-55 minutes: Slide 9: Pair-share process – 10 minutes to discuss in pairs, 15 minutes to debrief as a large group facilitated by the instructor
- 55-60 minutes: Slide 10: Class reads the Modern Hippocratic Oath out loud
- 60-85 minutes: Slides 11-12: Second pair-share task and orientation to the rest of the modules.
- 85-90 minutes: Slide 13: Evaluation (Appendix D)

**Experience with Implementation (Tips for Deployment)**

Experience to date with this module:

- See the notes pages from the slides for slide-by slide
- This module is intended to occur just before students enter the clerkship years. We found that the closer this can be scheduled to the actual start date of clerkships, the better, since the ideas are fresh in students’ minds when they enter clerkships. Also, if the session can be scheduled in close proximity to the start of clinical clerkships, the students typically are highly motivated to participate in the session. We schedule our session during a 5-day intersession that occurs the week before students enter their first core clerkship.
- The Harry Potter clip and the med student quote in the beginning of this session are particularly powerful; we have noticed that the room becomes very quiet immediately after these two triggers. While these triggers are powerful, we find it important to be mindful that both of these triggers are potentially negative ones, and can create significant levels of fear and/or stress for some students. We address this by using the two triggers to frame the session as “one to help students to *maintain* their own identity” through explicitly articulating ideals. We have found this framing to create high levels of engagement during the discussion activities.

Experience to date with the entire curriculum (4 modules implemented over 1 year):

- The curriculum is intended to be modular, meaning that with the exception of the first module (where students articulate their own practice ideals), the rest of the modules were designed to occur in any order at any point during the clinical years. At Penn State, we managed the logistics of the curriculum by “assigning” one module to each of three participating core clerkships (family medicine, internal medicine, and underserved medicine). This means that individual students experienced the modules in different orders and with different timings, depending on their individual clerkship schedules. Future work with this curriculum should explore the advantages and disadvantages of different models of delivery with respect to curricular context and timing. One of the disadvantages of our scheduling model is that some students had long periods of time between sessions, leading to extinguishment of some of the concepts of the curriculum; we are brainstorming ways of delivering the curriculum in more regular and predicable fashion in order to keep the ideas fresh in students’ minds.
- We purposely designed this curriculum to be ungraded and to not have any student assessments (other than required attendance). Our rationale for this decision was to foster free discussion of ideas, and to allow students to give voice to both positive and negative experiences, particularly in sessions where the core clerkship director was present (the clerkship directors were part of our pool of facilitators). For the most part, we found students to be very open and frank in their discussions. One of the downsides of this decision, though, was that participation was variable across students, with facilitators often estimating about 10% of students to have low engagement across sessions.
- While the modules in the curriculum have unique ways of fostering student discussion, all have a common overall structure of 15 minutes of individual writing followed by 45 minutes of group discussion. We chose to begin each session with a writing assignment for several reasons: a) we found that writing provided a good trigger for subsequent discussion, and b) the temporal relationship of writing immediately followed by discussion worked better (in terms of fostering discussion and not having to police the writing assignments) than requiring advance writing prior to coming to the session. We noticed that students who had access to writings that they had already done in the curriculum had enhanced participation in subsequent curriculum modules, however, most students either forgot or lost the writings from prior sessions. We therefore created an electronic folder in our course management system for each individual student (that only they could access), in order to keep their documents in an easily accessible and secure place. We reminded students to bring their laptops or internet-connected devices to the sessions so that they could complete and save the writing assignments into their unique folders.
- Some notes of facilitation of the modules in this curriculum:
  - Facilitators need to be comfortable with letting the conversation flow and probing students to deeper levels of reflection. They need to have skill in helping other students not to try to “fix” each others’ problems, but rather, to draw out the collective wisdom of the group or classroom.
  - We used a variety of facilitation models across the modules (e.g., clerkship director as facilitator, co-facilitation with hospital chaplains, multiple discipline-based facilitators, etc). Each model had strengths and drawbacks, but none emerged as better or worse than the others.
- We found time and space in the third-year clerkship curricula to be precious, so we had to work creatively with clerkship directors to protect time for these sessions. We found it useful to have clerkship directors on the planning committee for this curriculum in order to build trust and buy-in.

**Limitations, Prelim Evaluation Data, and Ideas for Improvement/Expansion**

Preliminary Evaluation Data for this module:

Evaluation of the curriculum took place in a traditional Likert format. Session evaluation averages on a 7 point Likert Scale (except for item 6, which used a 6-point scale) for 129 students:

1. Please rate how easy it was for you to reflect on your experiences in today’s session 4.80
2. Please rate the quality of your reflective process during today’s session 4.78
3. Please rate the usefulness of the individual assignment (writing) during today’s session 5.05
4. Please rate the usefulness of the group discussions during today’s session 4.62
5. Please rate the helpfulness of the faculty facilitator during today’s session 5.33
6. Please rate your agreement with this statement; “ I feel like I have new ideas to

try out after today’s session” 4.62

We conducted the session during an intersession week that occurred 1 week before students started clinical clerkships. The timing of the session was at the end of the day after approximately 6 hours of lectures that covered a variety of issues relating to clerkships (including HIPAA training, etc). Very few students wrote qualitative comments, and most focused on the problematic timing of the session:

- It'd be better if this session were held earlier in the day.
- Liked reviewing my ideas before starting rotations!
- The session would have been much better at the beginning of the day. Everyone was burned out by 4PM.
- This was a great idea, just really bad timing. I was too exhausted by the end of the day to reflect.
- I liked the structure of discussion. I wonder if there would be a way to make the self-reflection prompt shortened/more succinct.
- I think the session was great, but it would have been more appreciated in the morning.
- Should be streamlined to keep people engaged. I enjoyed the peer interaction.
- I greatly enjoyed the discussion w/entire group to get everyone's ideas rather than a small group. Session very well done & balanced.
- Session itself great idea; wonderful to implement; ratings reflect end of horrible 8-hr day.

There was also one comment that we feel demonstrates the reach of the clinical years’ hidden curriculum, even before the students had started ward rotations:

- This session was really well-intentioned, but honestly we're all more concerned about screwing up and killing someone rather than losing empathy.

Ideas for Improvement for the session and entire 4-module curriculum:

- Student evaluations suggest that the timing during the day for a reflection session is important.
- As noted above, we have not collected data on whether the order in which the modules are delivered makes a difference. Future work should address this issue.
- There also needs to be work to discern the best frequency and timing for the sessions, and whether additional topics are needed.
- Individual sessions tended to touch upon a wide variety of topics; future work to delineate the themes that are discussed during the modules would help to refine and position the curriculum.
- While student feedback has been generally positive, it is unclear whether the good experiences were due to the structure and focus of the course, or were due to just having protected time to discuss important experiences on the wards.
- Students consistently ranked the writing assignments as least helpful, while, in contrast, facilitators frequently observed high degrees of engagement during the individual writing activities and high quality discussion afterward. We elected, therefore, not to eliminate the writing portion of the modules.

**References**

1. Thompson BM, Teal CR, Rogers JC, Paterniti DA, Haidet P. Ideals, activities, dissonance, and processing: a conceptual model to guide educators’ efforts to stimulate student reflection. Acad Med 2010;85:902-908/
