# Supplementary material for: A Curriculum for Clerkship Students to Foster Professionalism Through Reflective Practice and Identity Formation
Source: MedEdPORTAL. 2016 Jun 17;12:10416. doi: 10.15766/mep_2374-8265.10416 (PMC6464454; doi:10.15766/mep_2374-8265.10416)
Supplement: Supplementary file 1 — A. Opening Session Articulating One's Ideals Facilitator's Manual.docx B. Opening Session Writing Prompt.docx C. Opening Session PowerPoint Slides.ppt D. Session Evaluation Form.docx E. Module 2 Facilitator's Guide.docx F. Module 3 Facilitator's Guide.docx G. Module 4 Facilitator's Guide.docx H. Module 4 Ideals Box Template.docx I. Module 4 Introductory Email With Table.doc [file mep-12-10416-s001.zip › B. Opening Session Writing Prompt.docx]

When you applied for medical school, you were asked to write a personal statement about why you wanted to join the profession of medicine. You may have also had conversations over the years with family, friends, teachers, mentors, and others about your aspirations for a career in medicine. Over the course of this time, you may have formed or be in the process of forming your own ideas and thoughts about what you want to “be like” as a physician.

Realizing that our own personal “vision” of what an ideal doctor is and does can help to anchor us in tough situations, today’s session is designed to help us gain clarity and specificity about what we want to be like as doctors.

For the next 15 minutes, please articulate your own personal vision for what you want to be like as a doctor. We would encourage as much creativity as possible, and to articulate this in the way that would be most helpful to you as you move through your clinical experiences in medical school. For example, you can write prose, a list, a poem, draw a picture, or use whatever verbal or visual medium has the most meaning for you personally.
