# Supplementary material for: A Curriculum for Clerkship Students to Foster Professionalism Through Reflective Practice and Identity Formation
Source: MedEdPORTAL. 2016 Jun 17;12:10416. doi: 10.15766/mep_2374-8265.10416 (PMC6464454; doi:10.15766/mep_2374-8265.10416)
Supplement: Supplementary file 1 — A. Opening Session Articulating One's Ideals Facilitator's Manual.docx B. Opening Session Writing Prompt.docx C. Opening Session PowerPoint Slides.ppt D. Session Evaluation Form.docx E. Module 2 Facilitator's Guide.docx F. Module 3 Facilitator's Guide.docx G. Module 4 Facilitator's Guide.docx H. Module 4 Ideals Box Template.docx I. Module 4 Introductory Email With Table.doc [file mep-12-10416-s001.zip › D. Session Evaluation Form.docx]

**PLEASE CIRCLE THE APPROPRIATE NUMBER:**

1. **Please rate HOW EASY it was to reflect on your experiences during today’s session:**

Not Easy Extremely

At All Easy

1 2 3 4 5 6 7

1. **Please rate the QUALITY of your reflective process during today’s session:**

Low Quality High Quality

1 2 3 4 5 6 7

1. **Please rate the usefulness of the INDIVIDUAL (e.g., writing) assignment during today’s session:**

Not Useful Extremely

At All Useful

1 2 3 4 5 6 7

1. **Please rate the usefulness of the GROUP DISCUSSIONS during today’s session:**

Not Useful Extremely

At All Useful

1 2 3 4 5 6 7

1. **Please rate the helpfulness of the FACULTY FACILITATOR during today’s session:**

Not Helpful Extremely

At All Helpful

1 2 3 4 5 6 7

1. **Please rate your agreement with this statement: “I feel like I have new ideas to think about after today’s session”**

Strongly Strongly

Disagree Agree

1 2 3 4 5 6

**We would LOVE to hear your feedback about today’s session. Please write any additional feedback or comments on the back of this page. Thank you SO MUCH!**
