# Supplementary material for: A Curriculum for Clerkship Students to Foster Professionalism Through Reflective Practice and Identity Formation
Source: MedEdPORTAL. 2016 Jun 17;12:10416. doi: 10.15766/mep_2374-8265.10416 (PMC6464454; doi:10.15766/mep_2374-8265.10416)
Supplement: Supplementary file 1 — A. Opening Session Articulating One's Ideals Facilitator's Manual.docx B. Opening Session Writing Prompt.docx C. Opening Session PowerPoint Slides.ppt D. Session Evaluation Form.docx E. Module 2 Facilitator's Guide.docx F. Module 3 Facilitator's Guide.docx G. Module 4 Facilitator's Guide.docx H. Module 4 Ideals Box Template.docx I. Module 4 Introductory Email With Table.doc [file mep-12-10416-s001.zip › E. Module 2 Facilitator's Guide.docx]

**Fostering Professionalism through Reflective Practice and Identity Formation Curriculum Module: When Your Ideals are Challenged Does Your Anchor shift?**

**Facilitator’s Guide**

This is one module of the “Fostering Professionalism through Reflective Practice and Identity Formation” Curriculum series. Apart from the initial module (Articulating Your Ideals), the curriculum has been designed to allow for implementation of modules in any order and over any time period.

In this guide, we present the module entitled “When Your Ideals are Challenged Does Your Anchor Shift?” which is presented during the Family and Community Medicine Clerkship. This module is designed to allow each student an opportunity to personally reflect on a professionalism issue encountered in the medical field. Students are then given the opportunity to discuss in dyads, using appreciative inquiry, the circumstances that challenged their belief system and why. By allowing time for a faculty-led facilitation at the end, it is hoped that students will be encouraged to reflect upon how the challenge has influenced their individual belief system, if at all, and how they might react differently in the future to a similar occurrence.

**Purpose and Goal of this Resource**

Purpose: To be able to express, through written reflection, how your ideals were challenged in the medical field and to apply appreciative inquiry to reconstruct the scenario for a colleague and as a nidus for faculty-facilitated group discussion.

Goals:

1. To identify an event when your professional ideals were challenged and reflect independently how your ideals were tested
2. To articulate, in both written and verbal form, the circumstance in which you felt conflicted
3. To examine how your ideals were challenged and be able to discuss with a colleague the scenario which led to your unease
4. To construct an analysis of your conflict and how you might adjust your reaction, if at all, to a similar scenario in the future if it were to occur
5. To encourage the student, through faculty facilitation, to summarize how they might react to similar challenges in the future

Conceptual background for this module: This module is designed to encourage students to reflect upon their core belief structure and to analyze the acculturating forces that may challenge those beliefs. By allowing students the opportunity to articulate how their prior ideals, individually attained and shaped through prior experiences and socialization, have been challenged in the medical setting, they will be able to explore alternative view points for future encounters. Such reflection may further strengthen a belief structure or allow mild modification in future encounters by an understanding of how others might view a similar scenario. In either case, such reflection will help the student better approach such occurrences in the future by allowing a review of the clinical situation from a personal and global viewpoint.

**Timeline and Practical Implementation Instructions**

Overall Session Structure (60 minutes): 10-15 third year medical students; one faculty facilitator

Initial Exercise (15 minutes):

Students are asked to write a self-reflection based upon the following cue:

“All of us have our own set of ideals to help guide us through personal and professional encounters. Recalling an experience that you have had in the medical field, take the next 15 minutes to articulate how your ideals were challenged by others. This scenario can be a patient scenario (where your ideal conflicted with the patient), a physician scenario (where your ideal conflicted with the attending/resident physician or another student), a professional scenario (your ideals are such that you believe an encounter was unprofessional), or a learning scenario (your ideals were challenged by a faculty member or another student).”

All reflections are available for student use only and not available to faculty. Students may elect to not share any of their reflections with other students or faculty.

Peer Exercise (15 minutes):

Students are asked to discuss their reflections, using appreciative inquiry, with a fellow student in dyads.

“We will spend the next 10-15 minutes discussing your reflections with a partner. Take time to set up the scenario to the best of your remembrance and present this to your colleague. What were the circumstances of the encounter? What specifically challenged your ideals? What was done that was consistent with your beliefs? How did others react – were you the only one who seemed to be conflicted? How did this encounter change your perspective OR help anchor your beliefs? Will your response/reaction to a similar situation in the future be any different? How might you challenge the other person (patient/fellow student/physician) in the future, if at all?”

Faculty-facilitated Discussion (20 minutes):

Faculty facilitator guidelines were distributed. Five faculty members have been trained and utilized as facilitators during the two years of this curriculum (24 sessions). Faculty members are encouraged to open up discussion to those students who wish to present their reflections/discussions. Faculty are asked to have other students participate and to encourage discussion about the experiences of other students in similar situations. Discussion points are to help encourage group reflection upon how they might react to a similar scenario in the future – in particular, how patient expectations (including the patient belief structure, health literacy and social context), professional expectations (including the hidden curriculum), and organizational challenges (financial considerations, insurance issues) become part of the greater dialogue.

Debriefing (10 minutes): Time is allotted to allow the students to give their input into the value of the session and as part of the overall curriculum. The individual and confidential nature of their written reflection is emphasized as well as the importance of allowing themselves time to be reflective about their medical experiences as a way to be able to view their ideals in the context of medical practice. Students are encouraged to contact the facilitator or clerkship director if the impact of the session requires further discussion given the potential influence on a given students’ ideals and beliefs.

**Experience with Implementation (Tips for Deployment):**

Experience to date with this module**:**

- Students have been very interactive within the framework of this module (see evaluations below). The ability of our facilitators, all of whom have additional training in communication skills and facilitation, to allow the students to have a safe environment to discuss their reflections in a voluntary manner has led to discussions that delineate the disconnect that often occurs between one’s ideals and “real world” decisions that are made. Student reflections are often heart-felt as they look to their peers and faculty for an open dialogue as to why things are done and how others might react. Students appreciate the time to discuss their quandaries with others and often come to the realization that they are “not alone” in questioning the behaviors, attitudes and actions of others to whom they are often junior in rank. Facilitation of the question about change in behavior or attitude in the future allows not only for the group dynamic to occur, but for the student to individually reflect to a “next time” scenario before it occurs.
