# Supplementary material for: A Curriculum for Clerkship Students to Foster Professionalism Through Reflective Practice and Identity Formation
Source: MedEdPORTAL. 2016 Jun 17;12:10416. doi: 10.15766/mep_2374-8265.10416 (PMC6464454; doi:10.15766/mep_2374-8265.10416)
Supplement: Supplementary file 1 — A. Opening Session Articulating One's Ideals Facilitator's Manual.docx B. Opening Session Writing Prompt.docx C. Opening Session PowerPoint Slides.ppt D. Session Evaluation Form.docx E. Module 2 Facilitator's Guide.docx F. Module 3 Facilitator's Guide.docx G. Module 4 Facilitator's Guide.docx H. Module 4 Ideals Box Template.docx I. Module 4 Introductory Email With Table.doc [file mep-12-10416-s001.zip › I. Module 4 Introductory Email With Table.doc]

Dear Students,

The PSUCOM Longitudinal Curriculum: Fostering Professionalism through Reflective Practice and Identity Formation has components embedded in the Underserved Medicine and Domestic Health Clerkship. At the beginning of this clerkship, you are asked to restate your personal vision statement of what an ideal doctor is. Then, throughout the clerkship, you are asked to populate a simple table (below), delineating the forces you encounter which are forces that help you sustain that ideal and forces which do not. Feel free to support your list with examples and stories and to expand the table. At times, you may even find that the same force may populate both categories!

| Sustaining Forces | Non-sustaining Forces |
| --- | --- |
|  |  |
|  |  |
|  |  |
|  |  |
|  |  |
|  |  |
|  |  |

On the final Friday of the clerkship, we will have a two hour reflective and interactive session with small group discussion. You will bring your completed table to the session, to facilitate discussion and completion of an individual and group exercise in reflection. **Also, please bring your laptop to facilitate completion of the personal reflective component.**

We look forward to this opportunity to work with you. Feel free to contact us with any questions.
